# Characterization, Antioxidant Activities, and Functional Properties of Mucilage Extracted from *Corchorus olitorius* L.

**DOI:** 10.3390/polym14122488

**Published:** 2022-06-18

**Authors:** Songmin Oh, Do-Yeong Kim

**Affiliations:** Department of Food Science and Biotechnology, Dongguk University-Seoul, 32, Dongguk-ro, Ilsandong-gu, Goyang-si 10326, Gyeonggi-do, Korea; dhthdals6@naver.com

**Keywords:** *Corchorus olitorius* L., mucilage, functional property, viscosity, emulsifying, antioxidant activity

## Abstract

This study extracted the mucilage from *Corchorus olitorius* L. to observe its chemical and functional properties and suggest its possible applications in various fields. *Corchorus olitorius* L. mucilage was isolated by hot water extraction. FT-IR and HPAEC-PAD were used to describe the chemical composition, and the functional properties and antioxidant activities of the mucilage were also examined. The mucilage was mainly composed of uronic acid (34.24%, *w*/*w*). The solubility was 79.48 ± 1.08% at 65 °C, the swelling index was 29.01 ± 2.54% at 25 °C, and the water-holding capacity and oil-binding capacity were 28.66 ± 1.48 and 8.423 ± 0.23 g/g, respectively. The mucilage viscosity increased from 4.38 to 154.97 cP in a concentration-dependent manner. Increasing the concentration decreased the emulsion activity and increased the emulsion stability, most likely because of the corresponding increase in surface tension and viscosity. Results from antioxidant assays confirmed that the in-vitro radical scavenging activity of the mucilage increased with concentration. This study shows that *C. olitorius* L. can be utilized as a new hydrocolloid source, with potential applications in fields ranging from foods to cosmetics and pharmaceuticals.

## 1. Introduction

Hydrocolloids are applied in various fields, such as biomedicine, bioengineering, and food technology. A hydrocolloid can be defined as a water-soluble polymer that affects viscosity and gelation [1]. Food hydrocolloids can be produced from a wide range of sources, such as plants, animals, synthetic chemicals, microorganisms, and seaweeds. In recent years, there has been a lot of research on the biopolymers extracted from by-products in the food industry using novel techniques [2,3]. Polysaccharide hydrocolloids are a highly active industrial domain because of their steadily increasing demand in various fields [4].

*Corchorus olitorius* is a green-yellow plant of the Tiliaceae family that grows naturally along the Mediterranean coast of Egypt in tropical and subtropical regions [5]. The plant is also known as molokhia, Egyptian spinach, mallow, Nalta jute, or Tossa jute [6]. *Corchorus olitorius* is widely used in Asia and Africa as a culinary and medicinal herb [6]. It is consumed in various forms with soup or meat in many African countries as the minerals, such as calcium and magnesium, and vitamins may contribute to preventing various adult diseases, and it contains a large amount of dietary fiber [7]. Although there are some debates regarding its nutritional value, *C. olitorius* is often cited as having a relatively high protein content, with all 10 essential amino acids [8]. In addition, the plant contains β-carotene, lutein, and phenolic substances, such as caffeoylquinic acid and quercetin, suggesting that it may provide antioxidant effects [9]. Moreover, it is well known that *C. olitorius* contains mucilage [10].

Mucilage is a water-soluble biopolymer with a high water-holding capacity (WHC) [11]. Structurally, it is a highly branched polysaccharide with various sugar and uronic acid molecules. Generally, it can be extracted from most plant seeds or leaves, such as *Pereskia aculeata* Miller, chia seed, and *Cereus triangularis* cladodes [12,13,14]. In particular, plant-derived mucilage has recently attracted much attention due to its distinctive physicochemical properties, and health and functional attributes. The utilization of mucilage is highly dependent on its unique functional properties and bioactive role [15]. The emulsifying [16], rheological properties [17], and strong suspending ability of mucilage are due to its ability to form colloidal liquid systems and hydrogels [18]. The viscosity of the gel can affect the texture of food. Therefore, mucilage can be used as a food additive, such as thickener, tablet binder, emulsifier, and emulsion stabilizer, in the manufacture of jelly, bakery, beverage, and ice cream [16,19], as a film-forming agent [20] and as a gelling agent [21].

To the best of our knowledge, the study of mucilage extracted from *C. olitorius* is still insufficient. Therefore, in this study, to explore its potential applications in various fields, the physicochemical (moisture/ash content, pH, zeta-potential and carbohydrate composition) and functional properties (solubility, swelling index, water-holding capacity, oil-binding capacity, viscosity, surface tension, emulsifying properties) of mucilage extracted from *C. olitorius* were investigated, and antioxidant activities of mucilage were also evaluated.

## 2. Materials and Methods

### 2.1. Materials

*Corchorus olitorius* leaves produced in Egypt were purchased from Dusonae Yackcho in Seoul, Korea, in a form that was powdered after drying and grinding the leaves without any treatment. Chemical reagents, including acetone, ethanol, methanol, arabic gum, xanthan gum, sodium dodecyl sulfate (SDS), and sodium carbonate, were purchased from Samchun (Seoul, Korea). Gallic acid, 2,2-diphenyl-1-picrylhydrazyl (DPPH), Folin-Ciocalteu’s phenol reagent, L-ascorbic acid, α-tocopherol, and 2,2′-azino-bis(3-ethylbenzothiazoline-6-sulfonic acid) (ABTS) were obtained from Sigma-Aldrich (St. Louis, MO, USA). All chemical reagents were of analytical grade.

### 2.2. Extraction of Mucilage

The mucilage in the *C. olitorius* leaves was extracted according to Jung et al. [22] with some modifications. The *C. olitorius* leaf powder was soaked in 20 volumes of distilled water and stirred at 50 °C for 2 h. The liquid extract was separated using a multi-layer muslin cloth bag. After the liquid extract was centrifuged at 2700 rpm for 20 min, the supernatant was collected. Ethyl alcohol was added to the supernatant up to a final concentration of 55% to precipitate the mucilage. Then, acetone was added to the precipitated mucilage in a quantity of two-times the volume of mucilage, and the mucilage was washed by shaking and centrifuging (2700 rpm, for 15 min) repeatedly to remove the chlorophyll. After that, the precipitated mucilage was dried at 40 °C for 2 h and then collected and ground using a blender. The mucilage extracted from *C. olitorius* L. was collected, and the yield was calculated according to Equation (1).

(1) $$\mathrm{Yield}\;(\%)=\frac{\mathrm{weight}\;\mathrm{of}\;\mathrm{dried}\;\mathrm{mucilage}}{\mathrm{weight}\;\mathrm{of}\;\mathrm{leaves}}\times 100$$

### 2.3. Characterization of Mucilage

#### 2.3.1. The Moisture Content and Ash Content

According to the Association of Official Analytical Chemists’ (AOAC) method 925.10 [23], the moisture content was calculated. Ash content was calculated following the AOAC method 923.03 [23].

#### 2.3.2. pH Determination

Mucilage solution was prepared at 0.25% (*w*/*v*), and the pH value was measured. The pH meter (ORION STAR A214, Thermo Fisher Scientific, Ward Hill, MA, USA) was calibrated using standard solutions of known pH (4, 7, and 10). Triplicate measurements of pH were recorded.

#### 2.3.3. Determination of Zeta Potential

The zeta potential of 0.1% (*w*/*v*) mucilage solution was measured in disposable, folded capillary cells (DTS 1070) using a zetasizer (Nano Series, Nano-ZS90, Malvern Instruments, Worcestershire, UK).

#### 2.3.4. Molecular Weight Distribution

The molecular weight of mucilage was analyzed using a gel permeation chromatography (GPC) system (HLC-8420GPC, Tosoh corporation, Tokyo, Japan) equipped with a refractive index detector. Chromatographic separation was achieved on TSK gel G2500PWxl-GMPWxl Columns (7.8 mm ID × 300 mm, Tosoh corporation) in combination with a PWxl guard column. The mobile phase was 0.1 M sodium nitrate solution at a flow rate of 1.0 mL/min. The molecular weight was calculated as the relative molecular weight using pullulan polysaccharide (Agilent Technologies, Inc., Santa Clara, CA, USA) as standards for calibration.

### 2.4. Analysis of Carbohydrate Composition

#### 2.4.1. Determination of Monosaccharides Composition

Two milligrams of the sample was hydrolyzed in 1 mL of 2 M trifluoroacetic acid at 121 °C for 2 h. A high-performance anion-exchange chromatography—pulsed amperometric detection (HPAEC-PAD) system (Thermo Fisher Scientific, Sunnyvale, CA, USA) equipped with a CarboPac™ PA1 column (4 × 250 mm; Thermo Fisher Scientific) was used to determine the monosaccharide content. Monosaccharide standards (galacturonic acid, glucuronic acid, rhamnose, galactose, arabinose, glucose, xylose, fucose, and mannose) were purchased from Sigma-Aldrich. The mobile phase was 18 mM NaOH for the neutral sugars and 150 mM NaOH for uronic acids. The flow rate of the mobile phase was kept constant at 1 mL/min, and the column was maintained at 35 °C.

#### 2.4.2. Fourier Transform-Infrared (FT-IR) Spectroscopy

Functional groups of the mucilage were determined by FT-IR spectroscopy. The Nicolet iS5 FT-IR spectrophotometer (Thermo Fisher Scientific) was equipped with an iD5 ATR accessory featuring a diamond crystal cell. FT-IR spectra were recorded over a wavelength range 3600–800 cm^−^^1^ at a resolution of 2 cm^−^^1^, with 16 scans per spectrum.

### 2.5. Functional Properties

#### 2.5.1. Solubility

Solubility was performed according to the method described by Alizadeh Behbahani et al. [24] with some modifications. Solutions of the mucilage (1.0%, *w*/*v*) were prepared in water and solvents (hexane and methanol) at different temperatures (25, 45, and 65 °C). The solutions were stirred at 300 rpm for 30 min and then centrifuged in a high-speed centrifuge (MF80, Hanil Science Ind., Seoul, Korea) for 30 min at 3000 rpm. Supernatants were collected and oven-dried at 100 °C for 12 h. The solubility was calculated according to Equation (2).

(2) $$\mathrm{Solubility}\;(\%)=\frac{\mathrm{Dry}\;\mathrm{weight}}{\mathrm{sample}\;\mathrm{weight}}\times 100$$

#### 2.5.2. Swelling Index

Swelling power was assessed following the method described by Keshani-Dokht et al. [25] with a slight modification. Solutions of the mucilage (1.0%, *w*/*v*) prepared at different temperatures (25, 45, and 65 °C) were centrifuged using a high-speed centrifuge (MF80, Hanil Science Ind.) for 30 min at 3000 rpm. The precipitated substance was then weighed. The swelling index was calculated by Equation (3).

(3) $$\mathrm{Swelling}\;\mathrm{index}\;(\%)=\frac{\mathrm{weight}\;\mathrm{after}\;\mathrm{centrifuge}}{\mathrm{sample}\;\mathrm{weight}\;\times \;(100\;-\;\mathrm{solubility})}\times 100$$

#### 2.5.3. Water-Holding Capacity (WHC) and Oil-Binding Capacity (OBC)

WHC and OBC were measured by methods modified from Thanatcha and Pranee [26]. For WHC, the mucilage was prepared at 1.0% (*w*/*v*) in distilled water, stirred for 30 min, and then centrifuged for 30 min at 3000 rpm in a high-speed centrifuge (MF80, Hanil Science Ind.). The supernatant was removed. The wet samples were weighed and WHC was calculated by Equation (4).

(4) $$\mathrm{Water}-\mathrm{holding}\;\mathrm{capacity}\;(g\;\mathrm{water}/g\;\mathrm{dry}\;\mathrm{sample}\;\mathrm{weight})=\frac{\mathrm{weight}\;\mathrm{of}\;\mathrm{wet}\;\mathrm{sample}\;-\;\mathrm{weight}\;\mathrm{of}\;\mathrm{dry}\;\mathrm{sample}}{\mathrm{weight}\;\mathrm{of}\;\mathrm{dry}\;\mathrm{sample}}$$

To measure OBC, the mucilage was prepared at 1.0% (*w*/*v*) in corn oil and mixed by vortex for 1 min. After keeping at room temperature for 30 min, the mixture was centrifuged for 30 min at 3000 rpm, and then the supernatant was removed. The oil-absorbed sample weight was recorded, and the OBC was calculated by Equation (5).

(5) $$\mathrm{Oil}\;\mathrm{absorption}\;(g\;\mathrm{oil}/g\;\mathrm{dry}\;\mathrm{sample}\;\mathrm{weight})=\frac{\mathrm{oil}\;\mathrm{absorbed}\;\mathrm{sample}\;\mathrm{weight}\;-\;\mathrm{weight}\;\mathrm{of}\;\mathrm{dry}\;\mathrm{sample}}{\mathrm{weight}\;\mathrm{of}\;\mathrm{dry}\;\mathrm{sample}}$$

#### 2.5.4. Viscosity

Viscosity was assessed by a method modified from Assi et al. [27]. Suspensions of various concentrations of the mucilage (0.05, 0.10, 0.15, 0.2, 0.5, 0.8, 1.0%, *w*/*v*), arabic gum (0.2, 0.5, 0.8, 1.0, 5.0, 10, 20%, *w*/*v*), and xanthan gum (0.05, 0.10, 0.15%, *w*/*v*) were stirred at room temperature for 6 h. Afterward, viscosity (cP) was evaluated using a viscometer (Brookfield DV-2T, Toronto, Canada) at a speed of 37 rpm.

#### 2.5.5. Surface Tension

Surface tension was determined according to the method described by Gebresamuel and Gebre-Mariam [28] with slight modifications. Mucilage solutions were prepared (0.01, 0.05, 0.2, 1.0%, *w*/*v*) and stirred at 80 °C for 1 h. The sample was placed in a vessel, and the tensiometer (Sigma 703D, KSV Instruments Ltd., Helsinki, Finland) recorded the surface tension by the du Noüy ring method.

#### 2.5.6. Emulsifying Activity Index (EAI) and Emulsifying Stability Index (ESI)

To determine the emulsifying property, stock solutions of mucilage and xanthan were prepared at 2.0% (*w*/*v*) by dispersing the powders separately in distilled water and then stirred continuously at 25 °C for 6 h. Both stock solutions were diluted to 0.2, 0.4, 0.6, 0.8, 1.0, and 1.5% (*w*/*v*).

EAI and ESI were calculated following the method modified by Hay et al. [29]. The solutions (0.2–1.5%, *w*/*v*) were centrifuged for 30 min at 3000 rpm using a high-speed centrifuge (MF80, Hanil Science Ind.). After removing the supernatant, the remaining solution was homogenized with 1 mL of corn oil in a conical tube using an HG-15D homogenizer (Daihan Scientific, Seoul, Korea) at 15,000 rpm for 3 min. Immediately after homogenization, to prevent any flocculation or adherence to the sides of the cuvette, a 40 μL aliquot of the homogenized solution was added to 4 mL of 0.1% SDS, and the absorbance was measured at 500 nm using a UV spectrometer (UV-1800, Shimadzu, Kyoto, Japan). The EAI was calculated by Equation (6).

(6) $$\mathrm{EAI}\;(m^{2}/g)=\frac{{2T\times A}_{0}\times \mathrm{dilution}\mathrm{factor}}{C\times \Phi \times 10,000}$$

where T = 2.303; $A_{0}$ = absorbance measured immediately after homogenization; dilution factor = 100; C = mass of emulsifier/volume (g/mL) of aqueous phase prior to emulsion formation; Φ is the oil volume fraction of the emulsion.

ESI was evaluated by a method similar to the EAI method. After 6 h, an additional 40 μL aliquot was treated as described above for EAI. The ESI was calculated using Equation (7).

(7) $$\mathrm{ESI}(h)=A_{0}\times \frac{\text{∆}t}{\text{∆}A}$$

where ∆t = 6 h; ∆A is the change in the absorbance measured at 0 h (A_0_) and 6 h (A_6h_).

### 2.6. Antioxidants Activity

#### 2.6.1. Determination of Total Phenol Content

The total phenol content was analyzed using the procedure proposed by Adetuyi and Dada [30]. The mucilage solution (1 mg/mL) was mixed with 2.5 mL of 10% Folin-Ciocalteu’s phenol reagent (*v*/*v*) and neutralized by 2.0 mL of 7.5% sodium carbonate. The mixture was incubated at 45 °C for 40 min, and then the absorbance was measured at 765 nm using a UV spectrometer (UV-1800, Shimadzu). The total phenol content was expressed as milligrams of gallic acid equivalents (mg GAE) per gram of dried mucilage.

#### 2.6.2. DPPH Free Radical Scavenging Assay

The assay by Adetuyi and Dada [30] was implemented. Firstly, 1 mL of the mucilage solution at 1 mg/mL was mixed with 4 mL of 0.1 mM DPPH methanolic solution. The tubes were shaken and kept in the dark for 20 min at room temperature. Then, the optical density (OD) of the mixture was measured at 517 nm using a UV spectrometer (UV-1800, Shimadzu). Controls were prepared in the same way, except that the sample was replaced with a reference compound (ascorbic acid and α-tocopherol). Free radical scavenging activity was calculated using Equation (8).

(8) $$\mathrm{Radical}\;\mathrm{scavenging}\;\mathrm{activity}\;(\%)=\frac{\mathrm{control}\;\mathrm{OD}\;-\;\mathrm{sample}\;\mathrm{OD}}{\mathrm{control}\;\mathrm{OD}}\times 100$$

#### 2.6.3. ABTS^•^^+^ Scavenging Assay

The ABTS^+^ radical scavenging activity of mucilage was determined according to the method described by Bayar et al. [31] with some modification. To produce ABTS^•^^+^, an equal amount of 7 mM ABTS stock solution was mixed with 2.45 mM potassium persulfate solution. The mixture was allowed to stand in a dark place at 0 °C for 12–16 h. The ABTS^•^^+^ solution was then diluted by ethanol until the absorbance was 0.700 ± 0.20 at 734 nm. For the test, 0.2 mL of the mucilage solution (1 mg/mL) was mixed with 1.8 mL of the ABTS^•^^+^ solution. After the mixture was kept for 10 min in a dark place, the absorbance was measured at 734 nm. A mixture of 0.2 mL ethanol and 1.8 mL of ABTS^•^^+^ solution was used as a control. Ascorbic acid (water-soluble) and α-tocopherol (fat-soluble) were used as reference compounds. The ABTS^•^^+^ scavenging activity was calculated by Equation (8) above.

### 2.7. Statistical Analysis

All tests were carried out in three replicates and the results were expressed as mean ± standard deviation (SD). Data were analyzed by one-way analysis of variance (ANOVA) using the SPSS 25.0 statistical software program (IBM Corp., Armonk, NY, USA). Deviations were considered significant at *p* < 0.05.

## 3. Results and Discussion

### 3.1. Characterization of Mucilage Extracted from Corchorus olitorius L.

#### Extraction Yield, pH, Proximate Analysis, and Zeta Potential

Table 1 shows the extraction yield, molecular weight, pH value, moisture, ash, zeta potential, and total phenol content of the mucilage extracted from *C. olitorius* L. The mucilage was precipitated by ethanol, and acetone was used to further remove the chlorophyll, which presented as a brown compound.

The yield of mucilage extracted from *C. olitorius* L. was 10.24% of the dry weight, a value higher than that reported in the literature for *C. olitorius* L. (8.05%) [22]. The molecular weight (Mw) of mucilage was determined to be 1.9 × 10^6^ Da. The Mw is comparable to mucilage extracted from flaxseed (1.5 × 10^6^ Da) [32] and *Abelmoschus esculentus* L. (2.1 × 10^6^ Da) [33], but higher than those reported by Jung et al. for *C. olitorius* L. (40 to 500 kDa) [22]. It is suggested that the difference in yield and molecular weight is a consequence of variations in the extraction, purification method, growing regions, varieties of raw materials, and analytical methods [34].

The pH value was slightly acidic due to the presence of uronic acid. The pH values are comparable to those reported by Contreras-Padilla et al. [35] for O. *ficus-indica* (5.5–6) but higher than those found by Monrroy et al. (4.8–5.0) [36]. The solution needs to be slightly acidic for optimal emulsion activity [36,37]. Fanyi et al. described that the mucilage had good emulsifying activity when the mucilage was slightly acidic or basic [38].

The moisture content was 9.04%, which was similar to the results of other studies that indicated 7.63% [39] and 10.5% [40]. The ash content was 11.69%. This was similar to some other studies that reported 15.58% [17] and 16.00% [41]. Furthermore, it was found that mucilage extracted from leaves was higher than from seeds; this was confirmed by comparing data from some other studies that reported 0.70% [42] and 5.60% [41].

The zeta potential was −44.03 mV, indicating that the mucilage solution had an overall negative charge. Kaewmanee et al. [43] reported that mucilage extracted from seven Italian cultivars of flax were from −9.25 to −19.8 mV.

### 3.2. Carbohydrate Composition Analysis

#### 3.2.1. Monosaccharides Composition

**Figure 1:**
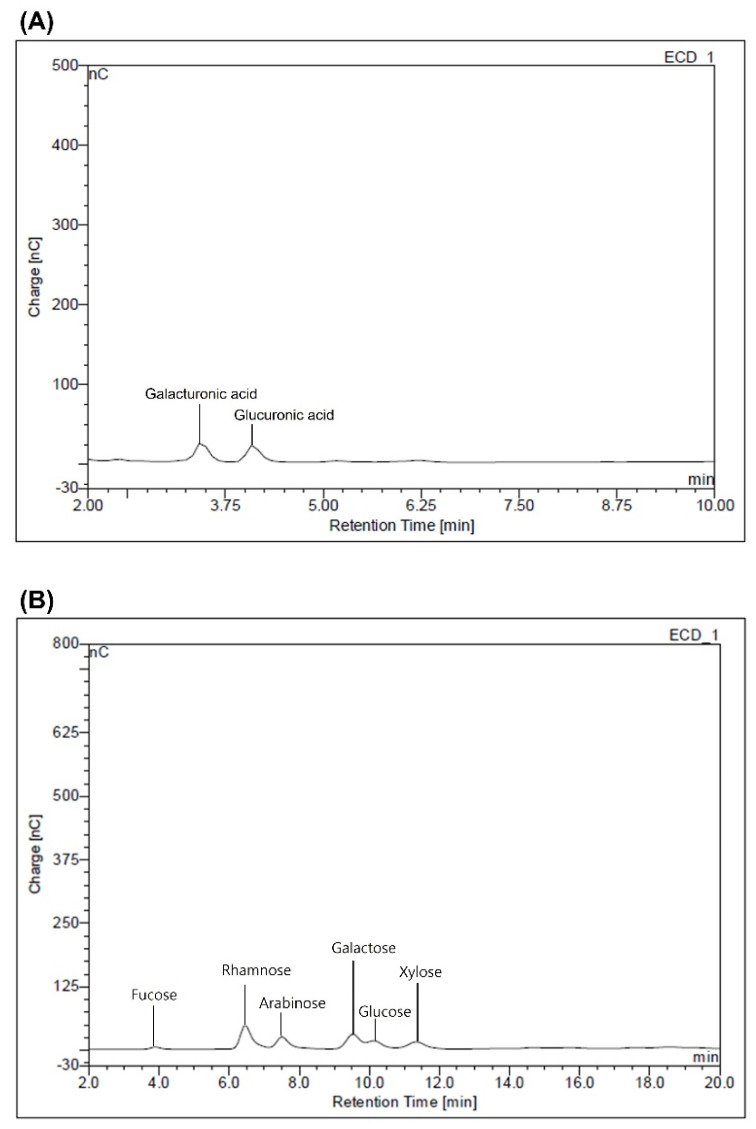
HPAEC-PAD chromatograms of the mucilage extracted from the *Corchorus olitorius* L. (**A**) uronic acid and (**B**) *neutral sugars*.

Table 2 shows the monosaccharide composition of the mucilage extracted from *C. olitorius* L. As shown in Figure 1, HPAEC-PAD chromatograms indicated that the monosaccharide composition qualitatively consists of six neutral sugars (rhamnose, xylose, galactose, glucose, arabinose, and fucose) and two uronic acids (glucuronic acid and galacturonic acid). Quantitatively, the major component in the mucilage structure was uronic acids, which accounted for about 34.3% of the total sugars, followed by rhamnose, galactose, arabinose, glucose, xylose, and fucose. This showed that the monosaccharide composition of mucilage extracted from leaves was similar to that published in other studies [17,44,45]. Hung and Lai [17] demonstrated that mucilage of *basella alba* has 4.38% of uronica acid, and arabinose (36.21%) and galactose (38.78%) were the main monosaccahrides, accounting for a comparative large proportion. In addition, Zeng and Lai [44] reported that uronic acid (17.1–21.3%) and glucose (24.4–36.0%) are the predominant monosaccharides in mucilage isolated from *asplenium australasicum*.

Generally, the leaf mucilage is largely composed of uronic acid and galactose, with relatively low amounts of xylose and fucose [46], and galactose and rhamnose are poorly represented in the monosaccharide composition of the seed mucilage [47]. However, various factors can influence the monosaccharide composition of plant-derived mucilage, such as the plant material, extraction conditions, cultivation environment conditions, or other compounds originating in the cellular wall or incomplete purification methods [39,45].

#### 3.2.2. Infrared Spectroscopy Analysis (FT-IR)

**Figure 2:**
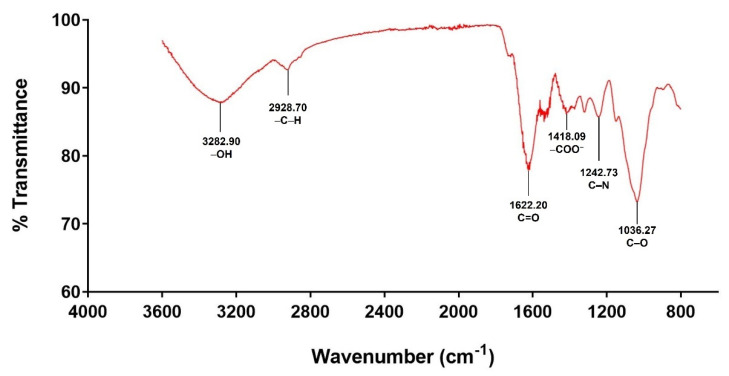
FT-IR spectrum of mucilage extracted from *Corchorus olitorius* L.

FT-IR spectroscopy was used to identify the functional groups present in mucilage and confirm the occurrence of peak characteristics of polysaccharides. The FT-IR spectrum for mucilage extracted from *C. olitorius* L. is shown in Figure 2. Hydroxyl (–OH) stretching vibration was represented by the broad band at 3282.90 cm^−1^. It indicated the presence of moisture in the sample due to the water-adsorption property in the polysaccharide [48]. The band at 2928.00 cm^−1^ was associated with the asymmetric vibration of –C–H bonds in methyl and methylene groups of monosaccharides [46,49]. The band at 1622.00 cm^−1^ could be ascribed to the asymmetric stretching of the C=O double bond in the carboxylic groups (–C=O^−^) [12,49], where the carboxyl groups interacted with some ionic bonds, affecting the viscosity and gel-forming property of hydrocolloids [50]. The peak at 1418.09 cm^−1^ was associated with the symmetrical deformation of the –C–H and carboxylate groups (–COO^−^) of acid residues [51,52]. The C–N amide III band at 1242.73 cm^−1^ arose from the acetyl groups of pectic residues [52]. In addition, it confirmed the presence of –OH phenol groups (water-soluble pigments, phenolic acids, coumarin derivatives) [46]. The band at 1036.27 cm^−1^ represented C–O ring vibrations of the main carbohydrates and could be associated with the presence of uronic acids [25].

### 3.3. Functional Properties

#### 3.3.1. Solubility

The solubility of mucilage evaluated at different temperatures in distilled water and at 25 °C in different solvents is reported in Table 3. As the temperature increased, the solubility of mucilage in distilled water increased significantly. Moreover, solubility was low in hexane and methanol compared to distilled water at different temperatures. There was no significant difference in the solubility between hexane and methanol. In other studies, researchers also confirmed that the mucilage was insoluble in the organic solvents [53,54]. This showed that mucilage extracted from *C. olitorius* L. possessed high polarity. Mucilage is an exopolysaccharide. It presents multiple –OH groups and hydrogen bonding, thereby inducing strong interaction between polysaccharide molecules and a strong affinity for water molecules [55]. According to Table 3, the mucilage extracted from *C. olitorius* L. had a similar solubility to the mucilage extracted from flaxseed (24.52–69.15% at 20–80 °C) [15].

#### 3.3.2. Swelling Index, WHC, and OBC

Examination of the swelling index at different temperatures revealed comparable indexes at 25 and 45 °C (29.01 ± 2.54 and 25.94 ± 2.62), but a significant decrease at 65 °C (19.51 ± 2.72). A high degree of swelling activity may lead to excessive hydration, reducing the interaction between mucoadhesive polymers due to low bioadhesiveness [56]. According to previous research, the swelling factor increased with the increase in pH and time [57,58].

The WHC of mucilage was 28.66 ± 1.48 g/g and its OBC was 8.42 ± 0.23 g/g. Mucilage in contact with water forms a three-dimensional network; it facilitates the ability of the mucilage to retain water and create a highly viscous solution. [36]. Moreover, the mucilage is a dietary fiber with a complex polysaccharide structure and a high water-absorption capacity and forms a gelatinous colloid by dissolving and dispersing in water [59]. Therefore, this water-soluble mucilage could greatly affect the texture of products in which it is incorporated [60]. Furthermore, in a previous study, Azubuike et al. [61] reported that the *C. olitorius* mucilage could be stable at a high temperature up to 300 °C through thermal anaysis by using differential scanning calorimetry. Due to its high water-absorption capacity and thermal stability, mucilage can be applied to foods, cosmetics, and pharmaceuticals [14]. The OBC reflects the ability of the mucilage to adsorb oil particles and inhibit the loss of flavor and oil from the food. According to our results, the mucilage extracted from *C. olitorius* L. displayed a similar OBC to guar and xanthan gum (4–6 g oil/g fiber) and, thus, could be useful as a food ingredient for maintaining flavor and improving taste in processed foods [42,62].

#### 3.3.3. Viscosity

**Figure 3:**
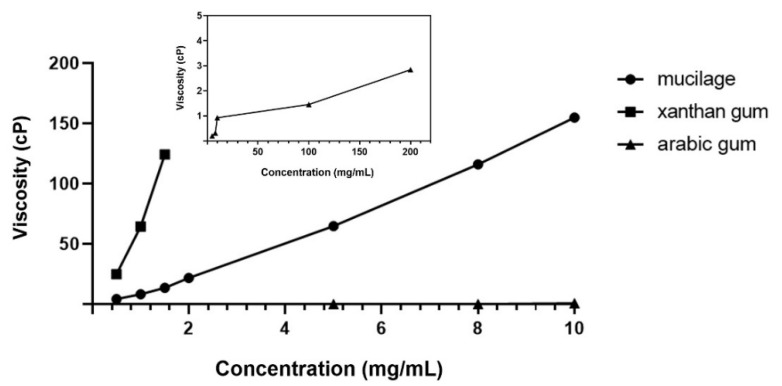
Viscosity of the mucilage from the *Corchorus olitorius* L., xanthan gum, and arabic gum at different concentrations. The inset represents the viscosity of the arabic gum at concentration of 5, 8, 10, 100, and 200 mg/mL.

Figure 3 shows the viscosity of mucilage, xanthan gum, and arabic gum. Viscosity increased dramatically with increased concentration of the samples, particularly for the mucilage compared to arabic gum, which showed similar values to those described by Monrroy et al. [36]. They proposed that viscosity increased from 0 to 317.50 cP (*Abelmoschus esculentus*), 515.50 cP (*Irvingia gabonensis*), and 801.93 cP (*Beilschmiedia mannii*), with increasing concentration of mucilage [27]. Figure 2 suggests that the mucilage solutions of 5.0 and 8.0 mg/mL have a similar viscosity to xanthan solutions of 1.0 and 1.5 mg/mL. Arabic gum and xanthan gum are widely applied alone or with other polysaccharides in many industries for a variety of purposes; in food applications as thickeners, binders, and stabilizers to increase the viscosity of food and improve the texture, and in non-food applications, such as synthetic perfumes and adhesives [63]. These results suggested a possibility of replacing food additives, such as xanthan gum and arabic gum, with the mucilage extracted from *C. olitorius* L.

#### 3.3.4. Surface Tension

**Figure 4:**
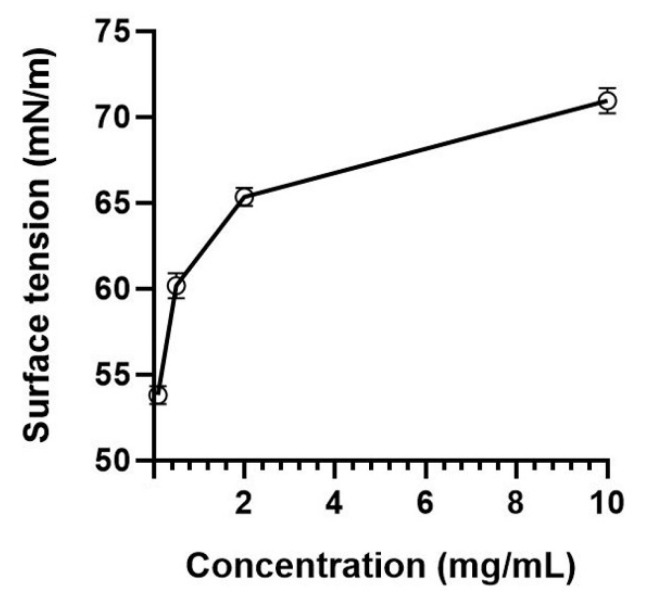
Surface tension of mucilage extracted from *Corchorus olitorius* L.

Mucilage showed a concentration-dependent increase in the surface tension, from 53.79 ± 0.53 mN/m (at 0 mg/mL) to 70.95 ± 0.73 mN/m (at 10 mg/mL), as shown in Figure 4. Naveed et al. [64] demonstrated that an increase in surface tension of mucilage concentrations above 1 mg/mL was probably an experimental artifact caused by the viscosity due to large polymers. They proposed the drop weight method to quantify surface tension rather than the du Noüy ring method, which does not consider the impact of viscosity [65]. Some systems, such as xanthan and carrageenan, show an increase in surface tension and interfacial tension with increasing concentration due to the gelation and viscosity at high concentrations [66,67].

#### 3.3.5. EAI and ESI

**Figure 5:**
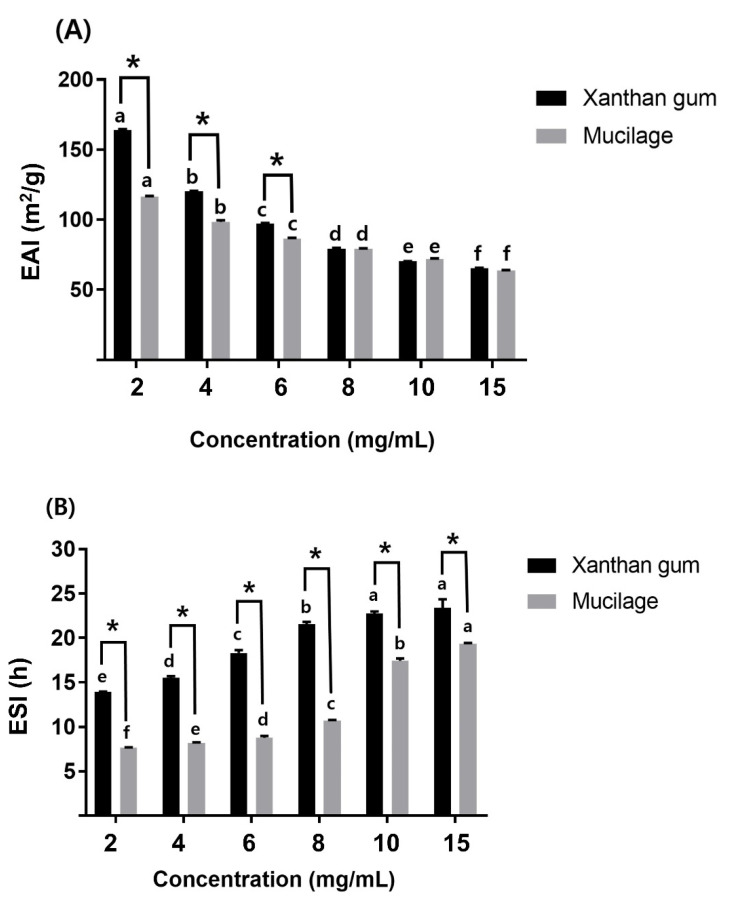
(**A**) Emulsifying activity index and (**B**) emulsifying stability index. Bars showing different labels (a–f) are significantly different (*p* < 0.05) in each sample at different concentrations. Bars labeled with an asterisk (*) are significantly different (*p* < 0.05) between the mucilage solution and xanthan gum solution at the same concentration.

The EAI of mucilage and xanthan gum decreased significantly with an increasing concentration (Figure 5A). There was a significant difference between the EAI of mucilage and xanthan gum up to 6 mg/mL, but no difference was found above 8 mg/mL. This was speculated to be due to the increased viscosity (Figure 3) and surface tension (Figure 4) with increasing concentration.

Satisfactory emulsion activity is achieved by lowering the surface and interfacial tension [68]. Another report showed that mucilage extraction by ethanol precipitation was the most suitable method to obtain high-quality mucilage powder with optimal emulsion properties compared to other methods [43]. Furthermore, it was suggested that the seed-derived mucilage increased EAI and decreased ESI, concentration dependently, as the surface tension decreased [42].

The results of the ESI evaluation of mucilage and xanthan gum revealed a significant difference between the two polysaccharides at every concentration examined, but the ESI of mucilage increased significantly with concentration (Figure 5B). Xanthan gum is classified as a polysaccharide with no or limited surface activity and gels or modifies the viscosity of the continuous aqueous phase to enhance the emulsion stability [69]. It is also used as an emulsion stabilizer due to its shear-thinning properties [70]. These features closely resembled those found in the mucilage in the current study, indicating that the mucilage extracted from *C. olitorius* L. and xanthan gum exhibited similar behaviors [10]. In addition, it was reported that because of the protein material intrinsic to polysaccharide hydrocolloids, the hydrocolloid primarily increased viscosity and acted to stabilize the emulsion [43]. This could explain the high emulsion stability of mucilage extracted from *C. olitorius* L., which, like xanthan gum, could be used as a stabilizer in food and pharmaceutical formulations.

### 3.4. Antioxidants Activity

**Figure 6:**
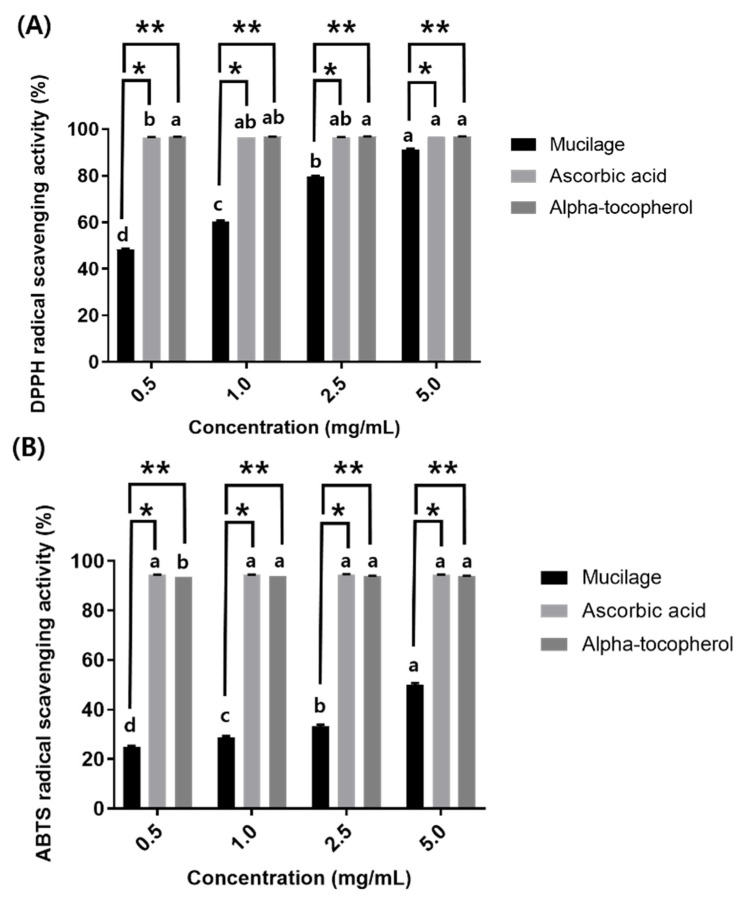
(**A**) DPPH free radical scavenging activity and (**B**) ABTS*^•^^+^* scavenging activity. Values of each bar with different labels (a–d) are significantly different (*p* < 0.05) in each sample at different concentrations. Bars labeled with a single asterisk (*) are significantly different (*p* < 0.05) between mucilage and ascorbic acid at the same concentration. Bars labeled with a double asterisk (**) are significantly different (*p* < 0.05) between mucilage and α-tocopherol at the same concentration.

Antioxidant potential was evaluated by measuring the free radical scavenging activity (DPPH and ABTS*^•^*^+^). These assays are widely used to investigate the antioxidant capacity of natural compounds [30,71]. In the DPPH assay, a lower absorbance indicates a higher DPPH free radical scavenging potential [72]. The DPPH (Figure 6A) and ABTS*^•^*^+^ (Figure 6B) antioxidant activity of mucilage were both lower compared to ascorbic acid (a water-soluble vitamin) and α-tocopherol (a fat-soluble vitamin) up to 2.5 mg/mL. With the increase in the concentration of mucilage, the antioxidant (DPPH and ABTS*^•^*^+^) activity increased dramatically, whereas ascorbic acid and α-tocopherol showed no change in either assay with increase in concentration. This phenomenon has already been observed in previous studies on the antioxidant activity of mucilage [12,31]. In addition, Jiang et al. [2] reported that LAB-derived EPSs could reduce the concentration of free radicals and acts as an excellent electron donor. In this study, the ABTS*^•^*^+^ radical scavenging activity supported the DPPH free radical scavenging activity. The results obtained in this work also showed a positive relationship between antioxidant activity and the phenol content (30.19 mg GAE/g) of mucilage (Table 1), indicating that the phenolic groups were strongly involved in the antioxidant capacity. These results demonstrated that mucilage could react effectively as an antioxidant by terminating the free radical chain reaction to form stable products, indicating that it could be an important natural antioxidant source [73].

## 4. Conclusions

Mucilage was extracted from *C. olitorius* L. by hot water extraction, with an average yield of 10.52%. It contained 9.04% moisture and 11.69% ash on a dry mass basis. The main monosaccharides were uronic acid, rhamnose, and galactose, and hydroxyl and carboxylic groups were the main functional groups. Solubility, swelling index, WHC, and OBC showed results that can contribute positively to food processing. In addition, its viscosity was equivalent to xanthan gum at low concentrations. Increasing the mucilage solution concentration decreased the emulsifying ability and increased the emulsifying stability due to the surface tension and viscosity effects. It was confirmed that the in-vitro antioxidant activity (DPPH and ABTS^•+^) of mucilage increased with concentration. Therefore, the mucilage obtained from *C. olitorius* L. showed physicochemical characteristics, antioxidant activity, and functional properties that allow it to compete with commercial hydrocolloids in various fields.

## Figures and Tables

**Table 1:** Results of yield, pH, and chemical composition of the mucilage extracted from the *Corchorus olitorius* L.

Parameters	Mucilage
Yield (%)	10.52 ± 0.39
Molecular weight (Da)	1.9 × 10^6^
pH	5.60 ± 0.01
Moisture content (%)	9.04 ± 0.38
Ash content (%)	11.69 ± 0.02
Zeta potential (mV)	−44.03 ± 2.53
Total phenol content (mg GAE/g of dried mucilage)	30.19 ± 0.23

Results are shown as mean with standard deviation from triplicate.

**Table 2:** Monosaccharide composition of the mucilage extracted from the *Corchorus olitorius* L.

Monosaccharide	Composition (%)
Rhamnose	23.8 ± 1.7
Galacturonic acid	18.8 ± 1.1
Glucuronic acid	15.5 ± 1.1
Galactose	14.0 ± 0.9
Arabinose	10.8 ± 0.6
Glucose	7.8 ± 0.3
Xylose	7.3 ± 0.5
Fucose	2.1 ± 0.1

Results are shown as mean with standard deviation from triplicate.

**Table 3:** Solubility of the mucilage from *Corchorus olitorius* L.

Solvent	Temperature (°C)	Solubility (%)
Distilled water	25	46.03 ± 0.79 ^cA^
	45	58.58 ± 1.83 ^b^
	65	79.48 ± 1.08 ^a^
Hexane	25	0.13 ± 0.03 ^B^
	45	0.26 ± 0.01 ^a^^B^
	65	0.31 ± 0.03 ^a^^B^
Methanol	25	0.35 ± 0.06 ^B^
	45	0.77 ± 0.03 ^b^^B^
	65	1.34 ± 0.02 ^a^^B^

Results are shown as mean with standard deviation. One-way analysis of variance (ANOVA) was carried out and means comparisons were executed by Duncan’s multiple range tests. Different superscripts (a,b,c) indicate significant differences (*p* < 0.05) among mucilage solution at different temperatures. Different superscripts (A,B) indicate significant differences (*p* < 0.05) among mucilage solution in different solvents at different temperatures (25, 45, 65 °C).

## Data Availability

The data presented in this study are available in the article.

## References

[1] Goff H.D., Guo Q. (2020). The role of hydrocolloids in the development of food structure. Handbook of Food Structure Development.

[2] Jiang B., Wang L., Zhu M., Wu S., Wang X., Li D., Liu C., Feng Z., Tian B. (2021). Separation, structural characteristics and biological activity of lactic acid bacteria exopolysaccharides separated by aqueous two-phase system. LWT.

[3] Wang Q., Liu W., Tian B., Li D., Liu C., Jiang B., Feng Z. (2020). Preparation and characterization of coating based on protein nanofibers and polyphenol and application for salted duck egg yolks. Foods.

[4] Soukoulis C., Gaiani C., Hoffmann L. (2018). Plant seed mucilage as emerging biopolymer in food industry applications. Curr. Opin. Food Sci..

[5] Loumerem M., Alercia A. (2016). Descriptors for jute (*Corchorus olitorius* L.). Genet. Resour. Crop Evol..

[6] Kuete V., Karaosmanoğlu O., Sivas H., Kuete V. (2017). Anticancer activities of african medicinal spices and vegetables. Medicinal Spices and Vegetables from Africa.

[7] Idirs S., Yisa J., Ndamitso M. (2009). Nutritional composition of *Corchorus olitorius* leaves. AJOL..

[8] El-Mahdy A.R., El-Sebaiy L.A. (1984). Preliminary studies on the mucilages extracted from okra fruits, taro tubers, jew’s mellow leaves and fenugreek seeds. Food Chem..

[9] Azuma K., Nakayama M., Koshioka M., Ippoushi K., Yamaguchi Y., Kohata K., Yamauchi Y., Ito H., Higashio H. (1999). Phenolic antioxidants from the leaves of *Corchorus olitorius* L. J. Agric. Food Chem..

[10] Yamazaki E., Kurita O., Matsumura Y. (2009). High viscosity of hydrocolloid from leaves of *Corchorus olitorius* L. Food Hydrocoll..

[11] Cui S.W., Nie S., Roberts K.T., Moo-Young M. (2011). Functional properties of dietary fiber. Comprehensive Biotechnology.

[12] Petera B., Delattre C., Pierre G., Wadouachi A., Elboutachfaiti R., Engel E., Poughon L., Michaud P., Fenoradosoa T.A. (2015). Characterization of arabinogalactan-rich mucilage from cereus triangularis cladodes. Carbohydr. Polym..

[13] Punia S., Dhull S.B. (2019). Chia seed (*Salvia hispanica* L.) mucilage (a heteropolysaccharide): Functional, thermal, rheological behaviour and its utilization. Int. J. Biol. Macromol..

[14] Silva S.H., Neves I.C.O., Oliveira N.L., de Oliveira A.C.F., Lago A.M.T., de Oliveira Giarola T.M., de Resende J.V. (2019). Extraction processes and characterization of the mucilage obtained from green fruits of pereskia aculeata miller. Ind. Crops Prod..

[15] Kaur M., Kaur R., Punia S. (2018). Characterization of mucilages extracted from different flaxseed (linum usitatissiumum l.) cultivars: A heteropolysaccharide with desirable functional and rheological properties. Int. J. Biol. Macromol..

[16] Ma F., Zhang Y., Yao Y., Wen Y., Hu W., Zhang J., Liu X., Bell A.E., Tikkanen-Kaukanen C. (2017). Chemical components and emulsification properties of mucilage from dioscorea opposita thunb. Food Chem..

[17] Hung P.-Y., Lai L.-S. (2019). Structural characterization and rheological properties of the water extracted mucilage of basella alba and the starch/aqueous mucilage blends. Food Hydrocoll..

[18] Nayak A.K., Pal D., Pany D.R., Mohanty B. (2010). Evaluation of spinacia oleracea l. Leaves mucilage as an innovative suspending agent. J. Adv. Pharm. Technol. Res..

[19] Lozano E., Salcedo J., Andrade R. (2020). Evaluation of yam (dioscorea rotundata) mucilage as a stabilizer in the production of mango nectar. Heliyon.

[20] Deore U.V., Mahajan H.S. (2018). Isolation and characterization of natural polysaccharide from cassia obtustifolia seed mucilage as film forming material for drug delivery. Int. J. Biol. Macromol..

[21] Tosif M.M., Najda A., Klepacka J., Bains A., Chawla P., Kumar A., Sharma M., Sridhar K., Gautam S.P., Kaushik R. (2022). A concise review on taro mucilage: Extraction techniques, chemical composition, characterization, applications, and health attributes. Polymers.

[22] Jung C.-H., Choi I.-W., Kim H.-M., Seog H.-M. (2002). Physicochemical properties of mucilage from domestic molokhia (*Corchorus olitorius*). Korean J. Food Sci. Technol..

[23] Horwitz W., International A. (2000). Official Methods of Analysis of Aoac International. Food Composition, Additives, Natural Contaminants Vol. 2.

[24] Alizadeh Behbahani B., Tabatabaei Yazdi F., Shahidi F., Hesarinejad M.A., Mortazavi S.A., Mohebbi M. (2017). Plantago major seed mucilage: Optimization of extraction and some physicochemical and rheological aspects. Carbohydr. Polym..

[25] Keshani-Dokht S., Emam-Djomeh Z., Yarmand M.-S., Fathi M. (2018). Extraction, chemical composition, rheological behavior, antioxidant activity and functional properties of cordia myxa mucilage. Int. J. Biol. Macromol..

[26] Thanatcha R., Pranee A. (2011). Extraction and characterization of mucilage in ziziphus mauritiana lam. Int. Food Res. J..

[27] Assi O.Y., Sidibe D., Konan Y.N.g., Coulibaly A., Mahan R.M., Biego H.M.G. (2017). Viscosity study of mucilages extracted from abelmoschus esculentus, beilschmiedia mannii, *Corchorus olitorius* and irvingia gabonensis from côte d’ivoire. J. Appl. Life Sci. Int..

[28] Gebresamuel N., Gebre-Mariam T. (2012). Comparative physico-chemical characterization of the mucilages of two cactus pears (*Opuntia* spp.) obtained from mekelle, northern ethiopia. J. Biomater. Nanobiotechnol..

[29] Hay W.T., Vaughn S.F., Byars J.A., Selling G.W., Holthaus D.M., Price N.P.J. (2017). Physical, rheological, functional, and film properties of a novel emulsifier: Frost grape polysaccharide from vitis riparia michx. J. Agric. Food Chem..

[30] Adetuyi F.O., Dada I.B.O. (2014). Nutritional, phytoconstituent and antioxidant potential of mucilage extract of okra (*Abelmoschus esculentus*), water leaf (*Talinum triangulare*) and jews mallow (*Corchorus olitorius*). Int. Food Res. J..

[31] Bayar N., Kriaa M., Kammoun R. (2016). Extraction and characterization of three polysaccharides extracted from opuntia ficus indica cladodes. Int. J. Biol. Macromol..

[32] Qian K.Y., Cui S.W., Wu Y., Goff H.D. (2012). Flaxseed gum from flaxseed hulls: Extraction, fractionation, and characterization. Food Hydrocoll..

[33] Xu K., Guo M., Du J. (2017). Molecular characteristics and rheological properties of water-extractable polysaccharides derived from okra (*Abelmoschus esculentus* L.). Int. J. Food Prop..

[34] Safdar B., Pang Z., Liu X., Jatoi M.A., Mehmood A., Rashid M.T., Ali N., Naveed M. (2019). Flaxseed gum: Extraction, bioactive composition, structural characterization, and its potential antioxidant activity. J. Food Biochem..

[35] Contreras-Padilla M., Rodríguez-García M.E., Gutiérrez-Cortez E., Valderrama-Bravo M.d.C., Rojas-Molina J.I., Rivera-Muñoz E.M. (2016). Physicochemical and rheological characterization of opuntia ficus mucilage at three different maturity stages of cladode. Eur. Polym. J..

[36] Monrroy M., García E., Ríos K., García J.R. (2017). Extraction and physicochemical characterization of mucilage from *Opuntia cochenillifera* (L.) miller. J. Chem..

[37] Daoub R.M.A., Elmubarak A.H., Misran M., Hassan E.A., Osman M.E. (2018). Characterization and functional properties of some natural acacia gums. J. Saudi Soc. Agric. Sci..

[38] Ma F., Wang R., Li X., Kang W., Bell A.E., Zhao D., Liu X., Chen W. (2020). Physical properties of mucilage polysaccharides from dioscorea opposita thunb. Food Chem..

[39] Bazezew A.M., Admassu Emire S., Teamir Sisay M., Kinyuru J. (2022). Extraction, phytochemical analysis, monosaccharide composition and functional properties of x. Americana seed mucilage. Bioact. Carbohydr. Diet. Fibre.

[40] Nikbakht Nasrabadi M., Goli S.A.H., Sedaghat Doost A., Roman B., Dewettinck K., Stevens C.V., Van der Meeren P. (2019). Plant based pickering stabilization of emulsions using soluble flaxseed protein and mucilage nano-assemblies. Colloids Surf. A Physicochem. Eng. Asp..

[41] Câmara A.K.F.I., Okuro P.K., Cunha R.L.d., Herrero A.M., Ruiz-Capillas C., Pollonio M.A.R. (2020). Chia (*Salvia hispanica* L.) mucilage as a new fat substitute in emulsified meat products: Technological, physicochemical, and rheological characterization. LWT Food Sci. Technol..

[42] Alpizar-Reyes E., Carrillo-Navas H., Gallardo-Rivera R., Varela-Guerrero V., Alvarez-Ramirez J., Pérez-Alonso C. (2017). Functional properties and physicochemical characteristics of tamarind (*Tamarindus indica* L.) seed mucilage powder as a novel hydrocolloid. J. Food Eng..

[43] Kaewmanee T., Bagnasco L., Benjakul S., Lanteri S., Morelli C.F., Speranza G., Cosulich M.E. (2014). Characterisation of mucilages extracted from seven italian cultivars of flax. Food Chem..

[44] Zeng W.-W., Lai L.-S. (2014). Characterization of the mucilage isolated from the edible fronds of bird’s nest fern (*Asplenium australasicum*). Food Hydrocoll..

[45] Sáenz C., Sepúlveda E., Matsuhiro B. (2004). *Opuntia* spp mucilage’s: A functional component with industrial perspectives. J. Arid. Environ..

[46] Faccio C., Machado R.A.F., de Souza L.M., Zoldan S.R., Quadri M.G.N. (2015). Characterization of the mucilage extracted from jaracatiá (carica quercifolia (a. St. Hil.) hieron). Carbohydr. Polym..

[47] Wu Y., Cui W., Eskin N.A.M., Goff H.D. (2009). Fractionation and partial characterization of non-pectic polysaccharides from yellow mustard mucilage. Food Hydrocoll..

[48] Adel A.M., El–Wahab Z.H.A., Ibrahim A.A., Al–Shemy M.T. (2010). Characterization of microcrystalline cellulose prepared from lignocellulosic materials. Part i. Acid catalyzed hydrolysis. Bioresour. Technol..

[49] Barka N., Abdennouri M., El Makhfouk M., Qourzal S. (2013). Biosorption characteristics of cadmium and lead onto eco-friendly dried cactus (opuntia ficus indica) cladodes. J. Environ. Chem. Eng..

[50] Pachuau L., Lalhlenmawia H., Mazumder B. (2012). Characteristics and composition of albizia procera (roxb.) benth gum. Ind. Crops Prod..

[51] Wang J., Somasundaran P. (2006). Mechanisms of ethyl(hydroxyethyl) cellulose–solid interaction: Influence of hydrophobic modification. J. Colloid Interface Sci..

[52] Brito A.C.F., Silva D.A., de Paula R.C.M., Feitosa J.P.A. (2004). Sterculia striata exudate polysaccharide: Characterization, rheological properties and comparison with sterculia urens (karaya) polysaccharide. Polym. Int..

[53] Ang A., Raman I. (2019). Characterization of mucilages from abelmoschus manihot linn., amaranthus spinosus linn. And talinum triangulare (jacq.) willd. Leaves for pharmaceutical excipient application. Asian J. Biol. Life Sci..

[54] Chaudhary A., Kulkarni G., Awasthi R., Kumar P. (2016). Investigation on binding properties of grewia asiatica mucilage in tablet formulations. Marmara Pharm. J..

[55] Kabir S.F., Rahman A., Yeasmin F., Sultana S., Masud R.A., Kanak N.A., Haque P., Naeem M., Aftab T., Khan M.M.A. (2022). Chapter one-occurrence, distribution, and structure of natural polysaccharides. Radiation-Processed Polysaccharides.

[56] Archana G., Sabina K., Babuskin S., Radhakrishnan K., Fayidh M.A., Babu P.A.S., Sivarajan M., Sukumar M. (2013). Preparation and characterization of mucilage polysaccharide for biomedical applications. Carbohydr. Polym..

[57] Elkhalifa A.E.O., Al-Shammari E., Adnan M., Alcantara J.C., Mehmood K., Eltoum N.E., Awadelkareem A.M., Khan M.A., Ashraf S.A. (2021). Development and characterization of novel biopolymer derived from abelmoschus esculentus l. Extract and its antidiabetic potential. Molecules.

[58] Ziemichód A., Wójcik M., Różyło R. (2019). Seeds of plantago psyllium and plantago ovata: Mineral composition, grinding, and use for gluten-free bread as substitutes for hydrocolloids. J. Food Process Eng..

[59] Del-Valle V., Hernández-Muñoz P., Guarda A., Galotto M.J. (2005). Development of a cactus-mucilage edible coating (*Opuntia ficus indica*) and its application to extend strawberry (*Fragaria ananassa*) shelf-life. Food Chem..

[60] Souza G.S., de Cassia Bergamasco R., Stafussa A.P., Madrona G.S. (2020). Ultrasound-assisted extraction of psyllium mucilage: Evaluation of functional and technological properties. Emir. J. Food Agric..

[61] Azubuike C.P., Alfa M.A., Oseni B.A. (2017). Characterization and evaluation of the suspending potentials of *Corchorus olitorius* mucilage in pharmaceutical suspensions. Trop. J. Nat. Prod. Res..

[62] Segura-Campos M.R., Ciau-Solís N., Rosado-Rubio G., Chel-Guerrero L., Betancur-Ancona D. (2014). Chemical and functional properties of chia seed (*Salvia hispanica* L.) gum. Int. J. Food Sci..

[63] Saha D., Bhattacharya S. (2010). Hydrocolloids as thickening and gelling agents in food: A critical review. J. Food Sci. Technol..

[64] Naveed M., Ahmed M.A., Benard P., Brown L.K., George T.S., Bengough A.G., Roose T., Koebernick N., Hallett P.D. (2019). Surface tension, rheology and hydrophobicity of rhizodeposits and seed mucilage influence soil water retention and hysteresis. Plant Soil.

[65] Kaully T., Siegmann A., Shacham D., Marmur A. (2007). The effect of viscosity on surface tension measurements by the drop weight method. J. Appl. Polym. Sci..

[66] Huang X., Kakuda Y., Cui W. (2001). Hydrocolloids in emulsions: Particle size distribution and interfacial activity. Food Hydrocoll..

[67] Lee B.-B., Chan E.-S., Ravindra P., Khan T.A. (2012). Surface tension of viscous biopolymer solutions measured using the du nouy ring method and the drop weight methods. Polym. Bull..

[68] Aryee A.N.A., Agyei D., Udenigwe C.C., Yada R.Y. (2018). Impact of processing on the chemistry and functionality of food proteins. Proteins in Food Processing.

[69] Bouyer E., Mekhloufi G., Rosilio V., Grossiord J.-L., Agnely F. (2012). Proteins, polysaccharides, and their complexes used as stabilizers for emulsions: Alternatives to synthetic surfactants in the pharmaceutical field?. Int. J. Pharm..

[70] Benmouffok-Benbelkacem G., Caton F., Baravian C., Skali-Lami S. (2010). Non-linear viscoelasticity and temporal behavior of typical yield stress fluids: Carbopol, xanthan and ketchup. Rheol. Acta.

[71] Zhou Y., Cui Y., Qu X. (2019). Exopolysaccharides of lactic acid bacteria: Structure, bioactivity and associations: A review. Carbohydr. Polym..

[72] Leong L.P., Shui G. (2002). An investigation of antioxidant capacity of fruits in singapore markets. Food Chem..

[73] Motiwala M.N., Dumore M.N., Rokde V.V., Bodhe M.M., Gupta R.A., Dumore N.G., Danao K.R. (2015). Characterization and antioxidant potential of coccinia indica fruit mucilage: Evaluation of its binding properties. Bioact. Carbohydr. Diet. Fibre.

